# Cecum axis (CecAx) preservation reveals physiological and pathological gradients in mouse gastrointestinal epithelium

**DOI:** 10.1080/19490976.2023.2185029

**Published:** 2023-03-05

**Authors:** Hannah M. Lunnemann, Nicolas G. Shealy, Michelle L. Reyzer, John A. Shupe, Emily H. Green, Uswah Siddiqi, D. Borden Lacy, Mariana X. Byndloss, Nicholas O. Markham

**Affiliations:** a Department of Veterans Affairs, Tennessee Valley Healthcare System, Nashville, TN, USA; b Epithelial Biology Center, Vanderbilt University Medical Center, Nashville, TN, USA; c Department of Medicine, Vanderbilt University Medical Center, Nashville, TN, USA; d Department of Pathology, Microbiology, and Immunology, Vanderbilt University Medical Center, Nashville, TN, USA; e Vanderbilt Institute for Infection, Immunology, and Inflammation, Vanderbilt University Medical Center, Nashville, TN, USA; f Mass Spectrometry Research Center, Vanderbilt University Medical Center, Nashville, TN, USA; g Department of Biochemistry, Vanderbilt University, Nashville, TN, USA

**Keywords:** Mouse modeling, infectious disease, cecum, *Clostridioides difficile*, *Salmonella enterica* serovar typhimurium, gastrointestinal infection

## Abstract

The mouse cecum has emerged as a model system for studying microbe-host interactions, immunoregulatory functions of the microbiome, and metabolic contributions of gut bacteria. Too often, the cecum is falsely considered as a uniform organ with an evenly distributed epithelium. We developed the cecum axis (CecAx) preservation method to show gradients in epithelial tissue architecture and cell types along the cecal ampulla-apex and mesentery-antimesentery axes. We used imaging mass spectrometry of metabolites and lipids to suggest functional differences along these axes. Using a model of *Clostridioides difficile* infection, we show how edema and inflammation are unequally concentrated along the mesenteric border. Finally, we show the similarly increased edema at the mesenteric border in two models of *Salmonella enterica* serovar Typhimurium infection as well as enrichment of goblet cells along the antimesenteric border. Our approach facilitates mouse cecum modeling with detailed attention to inherent structural and functional differences within this dynamic organ.

## Introduction

Mouse models are indispensable for studying gastrointestinal physiology and infection pathogenesis. The histological and cellular differences along both the small intestine and the colon have been characterized extensively and show remarkable variability with implications for gastrointestinal disease pathology.^1^ Accordingly, histological preparations of the mouse intestine and colon often involve the Swiss roll technique enabling comprehensive analysis of the entire bowel segment length in one section.^2^ The mouse cecum has recently been highlighted as a critical site for microbe-host-metabolite interactions,^3,4^ but much less is known about its histological topology. To our knowledge, no tissue preparation for the cecum has been described that is comparable to the Swiss roll.

The cecum collects intestinal contents from the small intestine before they pass into the colon. In rodents, the cecum is easily distinguished from that of humans because of its relatively large size. Some postulate the size of the cecum is directly proportional to an animal’s ability to ferment complex carbohydrates.^5^ In addition to nutritional functions, the cecum likely has a critical role in immune system maintenance and bacterial defense.^6,7^ Studies of *Clostridioides difficile* (*C. difficile*) infection using transoral spore-gavage in C57Bl/6J mice consistently show more epithelial damage and edema in the cecum than the colon or small intestine.^8,9^ These observations led us to question whether the mouse cecum contained pathological and/or physiological gradients across anatomic axes similar to the colon and small intestine.

We devised a simple and reproducible procedure called CecAx, for cecum axis preservation, to maintain the anatomic orientation of the mouse cecum for histopathological analysis. In the healthy mouse and during infection with either *C. difficile* or *Salmonella enterica* serovar Typhimurium (*S*. Tm), we observe gradients in tissue architecture, metabolism, and cell type. Specifically, we show that crypt depth is greatest at the ampulla and along the mesenteric border of the cecum. With imaging mass spectrometry, we demonstrate how metabolites and lipids have a gradation of abundance in healthy mice. During *C. difficile* infection, edema and inflammatory infiltration predominate at the mesenteric border of the cecum. However, goblet cells are enriched at the antimesenteric border in mice infected with *S*. Tm. The CecAx method enables careful comparisons across animals within the same anatomical regions of the cecum.

## Results

### Cecum axis (CecAx) preservation

**Figure 1.**
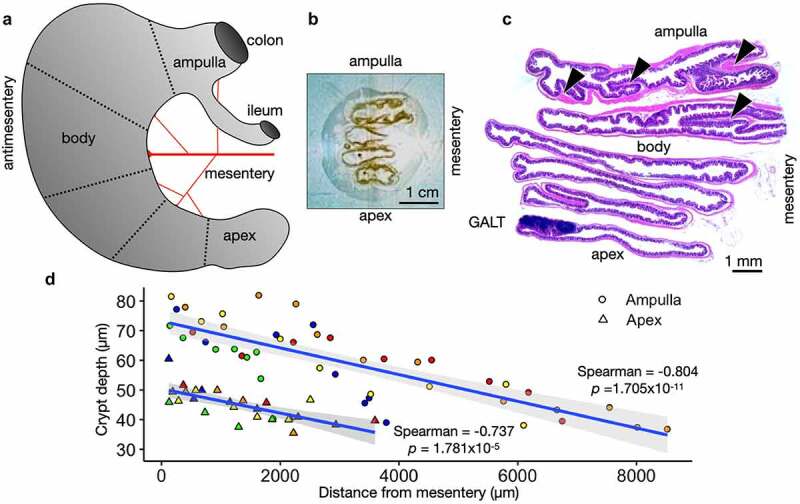
Cecum Axis (CecAx) preservation maintains both the ampulla-apex and mesentery-antimesentery axis and reveals histological gradients in epithelial crypt depth. A) Schematic of the mouse cecum with the ampulla, body, and apex of the cecum depicting the vascular bed attached to the mesenteric lesser curvature opposite from the antimesenteric greater curvature. B) Photograph of a faced tissue block showing formalin-fixed paraffin-embedded mouse cecum. C) Stitched brightfield microscopy images of H&E-stained mouse cecum show how the CecAx preservation technique yielded well-oriented tissue sections with respect to both epithelial crypts and organ anatomy. Arrowheads show examples of transverse folds. D) Individual measurements of crypt depth and distance from the mesentery in both the ampulla and apex of wild-type C57Bl/6J mice. The plot shows a negative correlation between depth and distance from the mesentery and shows that crypts were deeper in the ampulla compared to the apex. Crypts next to GALT are excluded (*n* = 5 mice; colors represent individual mice, Spearman’s rank correlation rho with p-value shown).

Our preparation of the mouse cecum maintains the anatomical orientation of the ampulla-apex axis and mesentery-antimesentery axis (Figure 1). The harvested, washed, and fixed mouse ceca were cut into pieces (dotted lines, Figure 1A), and the pieces were aligned with the cut edges down and the mesentery to the right (Figure 1B). The CecAx preservation method uniquely enables precise comparisons of specific regions within this large organ. Immediately, we observed the gut-associated lymphoid tissue (GALT) was not only found most commonly at the apex but always found at the antimesenteric side (Figure 1C), which has been previously described in the small intestine.^10^ Transverse folds were more numerous and longer toward the ampulla and mesenteric side (arrowheads, Figure 1C). We measured crypts from 5 wild-type C57Bl/6J adult mice and observed longer crypt depth in the ampulla compared to the apex. Furthermore, we observed longer crypt depth near the mesentery compared to the antimesentery (Figure 1D). These measurements did not include the crypts adjacent to GALT, which appeared much longer but often not completely visible in a single tissue section.

To determine if differences in proliferative cells account for the crypt depth gradient, we performed immunofluorescence and immunoblotting to detect Ki67+ cells and CyclinD1, respectively (Supp. Figure 1A-B). There were no discernable changes in these proliferation markers between the different cecal regions. To characterize tuft cell and enteroendocrine cell distribution, we stained wild-type C57Bl/6J mouse cecal tissues with antibodies against Dclk1 and ChromograninA, respectively. We observed more tuft cells at the antimesenteric border compared with the mesenteric border (Supp. Figure 2A-C) and increased enteroendocrine cells in the ampulla compared to the apex (Supp. Figure 2D-F).

**Figure 2.**
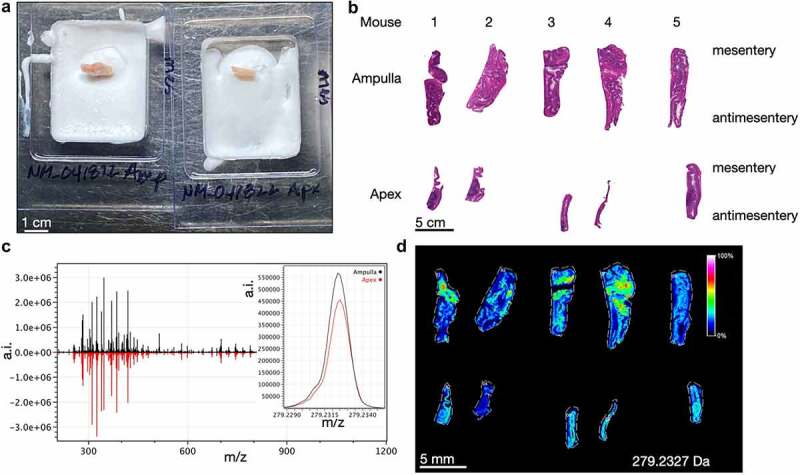
Imaging mass spectrometry of normal mouse cecum shows anatomic gradients of metabolites. A) the cecum ampulla (left) and apex (right) of a healthy adult C57Bl/6J mouse are shown after flash freezing and partial embedding in OCT. The mesenteric side is oriented to the right. B) Images of H&E stained serial mouse ceca sections with identical orientation to the sections imaged via imaging mass spectrometry in D. C) Averaged spectral intensities for 5 ampulla (black, top) and 5 apices (red, bottom). The inset shows a narrow portion of the averaged spectra for 279.233 Da. D) Heatmap showing spatial distribution of the intensity of a signal at m/z 279.233 Da, which putatively represents linoleate. a.i. = arbitrary intensity, m/z = mass/charge.

### Metabolic gradients revealed by imaging mass spectrometry

To determine if these histological gradients correlate with physiological differences, we chose the unbiased approach of imaging mass spectrometry for metabolites and lipids. We were inspired by a similar approach recently performed using gerbil stomachs to study *Helicobacter pylori*.^11^ Wild-type, adult C57Bl/6J mouse ceca were cut into pieces as in Figure 1. Ampulla and apex pieces were flash frozen before partial embedding in optimal cutting temperature media (Figure 2A). This technique enables unfixed, unembedded tissues to be sectioned onto a single slide for matrix application and imaging.^11^ Additional H&E-stained sections were obtained post-imaging alignment (Figure 2B). The mass spectral intensities (arbitrary intensity, a.i.) from each ampulla piece were averaged and compared to the averaged spectra from apex pieces (Figure 2C). Overall, the signal intensities and distributions are similar, however there are unique differences that may be related to tissue region. One example is the signal at m/z 279.233 Da, tentatively identified as linoleate via accurate mass, which has a higher signal in the ampulla compared to apex tissues. Linoleate is a medium-chain unsaturated omega-6 fatty acid implicated in infectious and inflammatory colitis (Figure 2C inset and 2D).^12,13^ The distribution for this lipid is predominantly epithelial with highest accumulation toward the mesentery. The full spectra for each of the 10 tissues are shown in Figure 2B, and the raw data are available in Supplementary Table 1 (ampulla tissues) and Supplementary Table 2 (apex tissues).

### Edema and inflammation are greater at the mesentery in C. difficile infection

**Figure 3.**
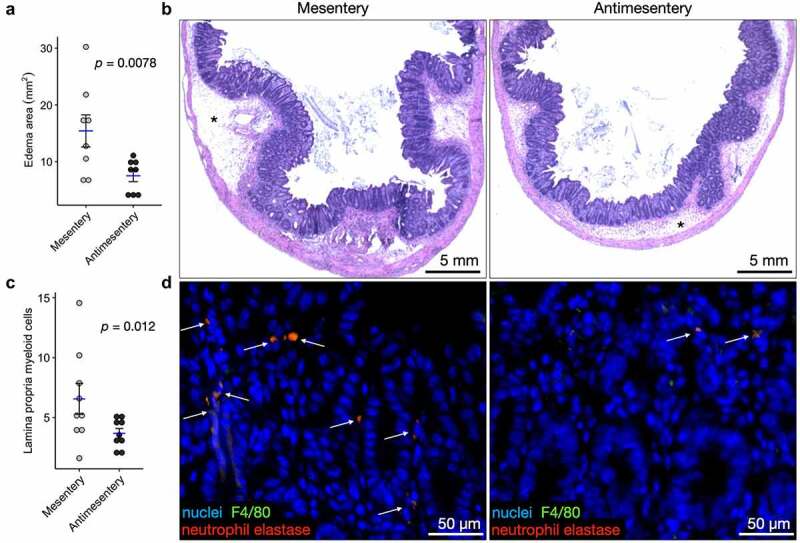
Difficile infection induces a gradient of edema and inflammation along the mesentery-antimesentery axis. A) Quantification of edema during *C. difficile* infection. Each point represents the average edema area at the mesentery and antimesentery from one mouse, *n* = 8. B) H&E images depicting edema area (asterisks, *) at the mesenteric and antimesenteric border. C) Quantification of inflammatory infiltration as measured by the number of myeloid cells (predominantly neutrophils) in the lamina propria per HPF (20×). Each point represents the average number of lamina propria myeloid cells at the mesentery and antimesentery from one mouse, *n* = 8. D) Immunofluorescence microscopy images show double-positive (orange represents the colocalization of F4/80 and neutrophil elastase staining) myeloid cells in the lamina propria. p-values shown from paired Wilcoxon signed-rank exact test.

We questioned whether any histological differences were present during disease. Mice infected with *C. difficile* had ceca that were consistently more edematous toward the mesentery compared to the antimesentery (Figure 3A-B). Edema was not observed under or next to GALT in any mouse. Inflammation was measured by the number of myeloid cells in the lamina propria, and average cells per high-power field (HPF) were higher at the mesentery compared to the antimesentery (Figure 3C-D). We observed myeloid cells as having double-positive staining by immunofluorescence for antibodies against F4/80 and neutrophil elastase. Based on review of the H&E images, the majority of these myeloid cells had a polymorphonuclear appearance.

### Opposite distribution of edema and goblet cells in S. tm infection

**Figure 4.**
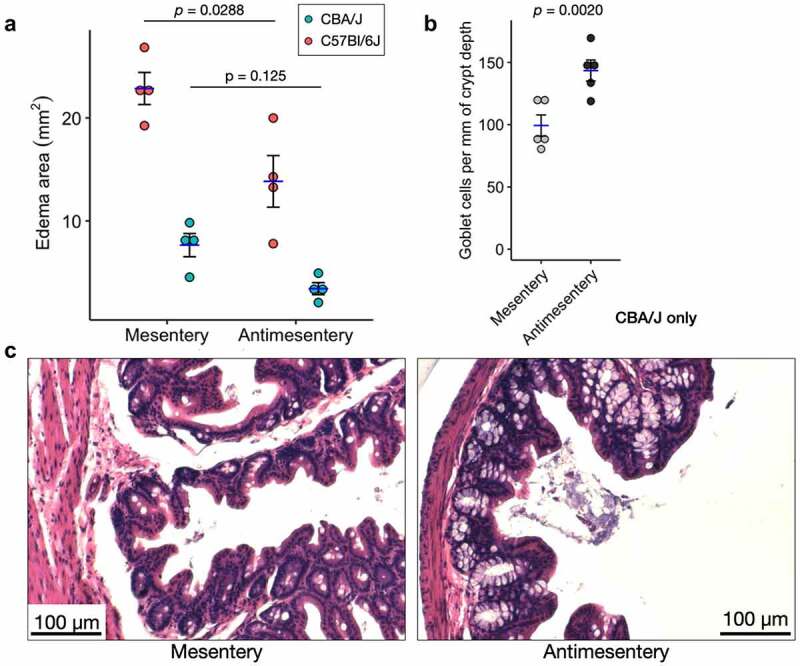
Two different S. Tm infection models show differential pathology along the mesentery-antimesentery axis. A) Quantification of edema area as measured in Figure 3 for C57Bl/6J and CBA/J models of S. Tm infection, *n* = 4 mice for each model. p-values are shown from a paired, two-tailed T-test. B) Quantification of goblet cell number shows higher average abundance at the antimesentery compared to mesentery of CBA/J mice, *n* = 5 mice. Because crypt depths are variable, we normalized the number of goblet cells per mm of their resident crypt depth. p-value shown from paired Wilcoxon signed-rank exact test. **C**) H&E images from S. Tm-infected CBA/J mice depicting differences in goblet cell density.

To determine the generalizability of these pathological gradients, we examined the same parameters in both C57Bl/6J and CBA/J genetic backgrounds of mice infected with *S*. Tm. It is well known that *S*. Tm preferentially affects M cells in the follicle-associated epithelium on GALT.^14,15^ To the best of our knowledge, no reports have described other histopathological differences with respect to the cecum axes in this infection. As was the case with *C. difficile*, we see greater edema at the mesenteric side compared to the antimesentery after *S*. Tm infection. While both models showed an apparent difference, only the C57Bl/6J model was statistically significant (Figure 4A). In the CBA/J model, but not C57Bl/6J, we observed more goblet cells per mm of crypt depth along the antimesentery (including crypts near GALT) compared to the mesentery (Figure 4B-C).

## Discussion

CecAx preservation maintains the ampulla-apex axis and mesentery-antimesentery axis for histopathological analysis of the mouse cecum (Figure 1). The preparation method can be applied to other rodent models and potentially the human appendix. There is no consensus agreement on the essential function of the mouse cecum or the human appendix. Hypotheses for their function range from immune regulation to microbiome maintenance to anaerobic fermentation.^16^ While our method alone does not resolve this question, it emphasizes the importance of spatial relationships in the gastrointestinal system as critical parameters for understanding host physiology and bacterial infection. Furthermore, CecAx preservation is similar to the intestine Swiss roll for avoiding the potential pitfall of comparing different cecal regions across mice and drawing false conclusions.

In gastrointestinal physiology and pathology, increased crypt depth is often correlated to increased proliferation rate.^17^ Our experiments show a two-fold difference in crypt depth between the mesenteric ampulla and antimesenteric apex (Figure 1D). However, we do not observe proliferation differences between cecal regions. Perhaps at earlier stages of development, mice might exhibit region-specific proliferative changes. Alternatively, there might be different rates of cell death accounting for the crypt depth gradient. We detect gradients of tuft cell and enteroendocrine cell abundance. The predominance of tuft cells along the antimesenteric border may be a reflection of their apparent enrichment near GALT (Supp. Figure 2A). Increased IL-13 from group 2 innate lymphoid cells induces tuft cell differentiation,^18^ which could be a mechanism underlying our observation.

Using the unbiased approach of imaging mass spectrometry, we have created a spectral map for putative metabolites and lipids in the adult wild-type C57Bl/6J mouse cecum. This resource is available in the Supplementary Material (Supp. Tables 1 & 2). A signal at m/z 279.233, tentatively corresponding to linoleic acid (18:2), was found to be differentially expressed in the mouse cecal epithelium (Figure 2D). It is an essential omega-6 fatty acid precursor for arachidonic acid, which is converted by cyclooxygenase enzymes into prostaglandins and eicosanoids with a wide range of homeostatic and immunoregulatory functions in the gut.^12^ And, it illustrates how functional molecules are unevenly distributed along gradients in the ampulla-apex axis and mesentery-antimesentery axis.

Multiple models of *C. difficile* infection have identified the mouse cecum as the organ with the highest bacterial load and pathological tissue injury.^9,19^ Here, we used CecAx preservation to carefully characterize the edema and acute inflammatory response with respect to cecal anatomy. The increased edema and myeloid cell infiltration at the mesentery compared to the antimesentery might be related to the location of bacteria in the lumen or host tissue. It could also be a result of the proximity to more vasculature. The mouse cecum is vascularized via a branch from the superior mesenteric artery. Smaller branches from the same main artery supply the proximal colon and distal ileum.^5^ Mechanistic experiments to test these hypotheses and determine cause-effect relationships are a part of more complex, ongoing studies.

Similar to *C. difficile* infection, examination of the *S*. Tm-infected mouse ceca shows increased edema at the mesentery compared to the antimesentery. This observation demonstrates the robustness of the CecAx preservation method and the consistency of the differential edema phenotype. Interestingly, *S*. Tm preferentially infects follicle-associated epithelium found along the antimesenteric boarder exclusively, but the edema is most pronounced at the mesenteric boarder. This relationship suggests the vascular location may have a greater impact on the amount of edema than the location of infection. In the CBA/J mouse model of *S*. Tm infection, the enrichment of goblet cells along the antimesentery demonstrates the existence of opposing gradients along this axis. It is not clear if there is a functional relationship between edema and goblet cells.

In summary, we show a simple and robust method for CecAx preservation. We also present evidence for why careful attention must be given to cecal anatomy when examining differences in proliferation, metabolite abundance, inflammation, edema, or cell type distribution.

## Materials and methods

### Animals

All mice were maintained in an AAALAC-accredited facility and procedures were performed using protocols approved by the Institutional Animal Care and Use Committee. All mice were purchased from The Jackson Laboratory. *C. difficile* infections were performed as described previously.^8^ Specifically, C57Bl6/J mice were challenged with either 1 × 10 spores or 1 × 10 spores from the R20291 strain and euthanized 4 days or 2 days after infection, respectively. *S*. Tm infections were performed as described previously.^20^ Specifically, C57BL/6J mice were given 5 g/L streptomycin in the drinking water on day zero. Regular water was given on day 2 followed by infection with *S*. Tm IR715 (1 × 10^9^ CFU) on day 3. Mice were euthanized on day 6, and cecal tissue was collected. CBA/J mice were orally inoculated with 1 × 10^9^ CFU of *S*. Tm IR715 without any pre-treatment. Five days after infection, ceca were collected for formalin fixation and paraffin embedding. Facilities are regularly tested for pathogens and stocked with clean bedding and free access to food and water. Mice had 12-h cycles of light and dark.

### CecAx preservation

To maintain the orientation of each axis, ceca were harvested, washed, and fixed with careful attention to their anatomic regions. Ampulla, body, and apex pieces were cut in the same fashion as depicted in Figure 1. The cut side was always placed down into a cryomold or tissue cassette for flash freezing or paraffin embedding, respectively. The mesentery was always aligned to the right side and labeled. Confirmation of the mesenteric orientation was typically possible with microscopic visualization of large vessels attached to the serosal surface (seen in the cecal body pieces of Figure 1C).

### Tissue staining and imaging

Tissue was sectioned at 5 μm thickness onto Superfrost Plus microscopy slides (Fisher Scientific). Deparaffinization, rehydration, H&E staining, and immunofluorescence were performed as previously described.^21^ Antibodies for immunofluorescence were anti-neutrophil elastase-AF555 (#bs-6982 R-A555, Bioss USA), anti-Ki67 (#652402, BioLegend), anti-chromograninA-Cy7 (#sc393941, SantaCruz), anti-Dclk1-Cy3 (#ab187184, Abcam), anti-CyclinD1 (#ab134175, Abcam), and anti-F4/80 (#MF48000, ThermoFisher) with anti-rat-488 (#405418, BioLegend) or anti-rat 555 (#405420, BioLegend). Images were obtained on Keyence BZ-X800 or Leica EVOS microscopes. Tissue area and histology measurements were performed in ImageJ2.^22^

### Imaging mass spectrometry

C57Bl/6J wild-type adult mouse ceca were harvested and washed in ice cold phosphate-buffered saline. Tissue was flash frozen and partially embedded in OCT compound (Tissue-Tek). Tissue was sectioned at 12 μm thick on a cryostat (Leica Biosystems). MALDI matrix (9-aminoacridine, 9AA) (Sigma-Aldrich) was spray-coated onto the target slides in an automated fashion using a TM Sprayer (HTX Imaging). 9-AA was made up as a 5 mg/ml solution in 90% methanol. Four passes were used with a nozzle temperature of 85°C, a flow-rate of 0.15 ml/min, 2-mm track spacing, and a stage velocity of 700 mm/min. Nitrogen was used as the nebulization gas and was set to 10 psi. Images were acquired on a 15T Fourier transform ion cyclotron resonance mass spectrometer (FT-ICR MS, Solarix, Bruker Daltonics) equipped with an Apollo II dual ion source and Smartbeam II 2 kHz Nd:YAG laser that was frequency tripled to 355 nm. Data were collected in the negative ion mode with the laser operating at 2 kHz at 75 μm resolution. Tentative metabolite identifications were made by accurate mass, typically better than 1 ppm. Images were analyzed with flexImaging software (Bruker), while average spectra were exported to mMass for visualization of differences.^23^

### Statistical analysis

Data were plotted and tested for statistical significance using the following packages: ggplot2, ggpubr, rstatix, and tidyverse in R version 3.6.3.^24–28^ Spearman’s rank correlation was performed for crypt depth and distance from the mesentery. The paired Wilcoxon signed rank exact test was used to compare two groups from non-parametric data, and the paired T-test was used to compare two groups from parametric data. Statistical significance was defined as *p* < 0.05.

## Supplementary Material

Supplemental Material
