# Supplementary figures and images for: Cecum axis (CecAx) preservation reveals physiological and pathological gradients in mouse gastrointestinal epithelium
Source: Gut Microbes. 2023 Mar 5;15(1):2185029. doi: 10.1080/19490976.2023.2185029 (PMC10012889; doi:10.1080/19490976.2023.2185029)

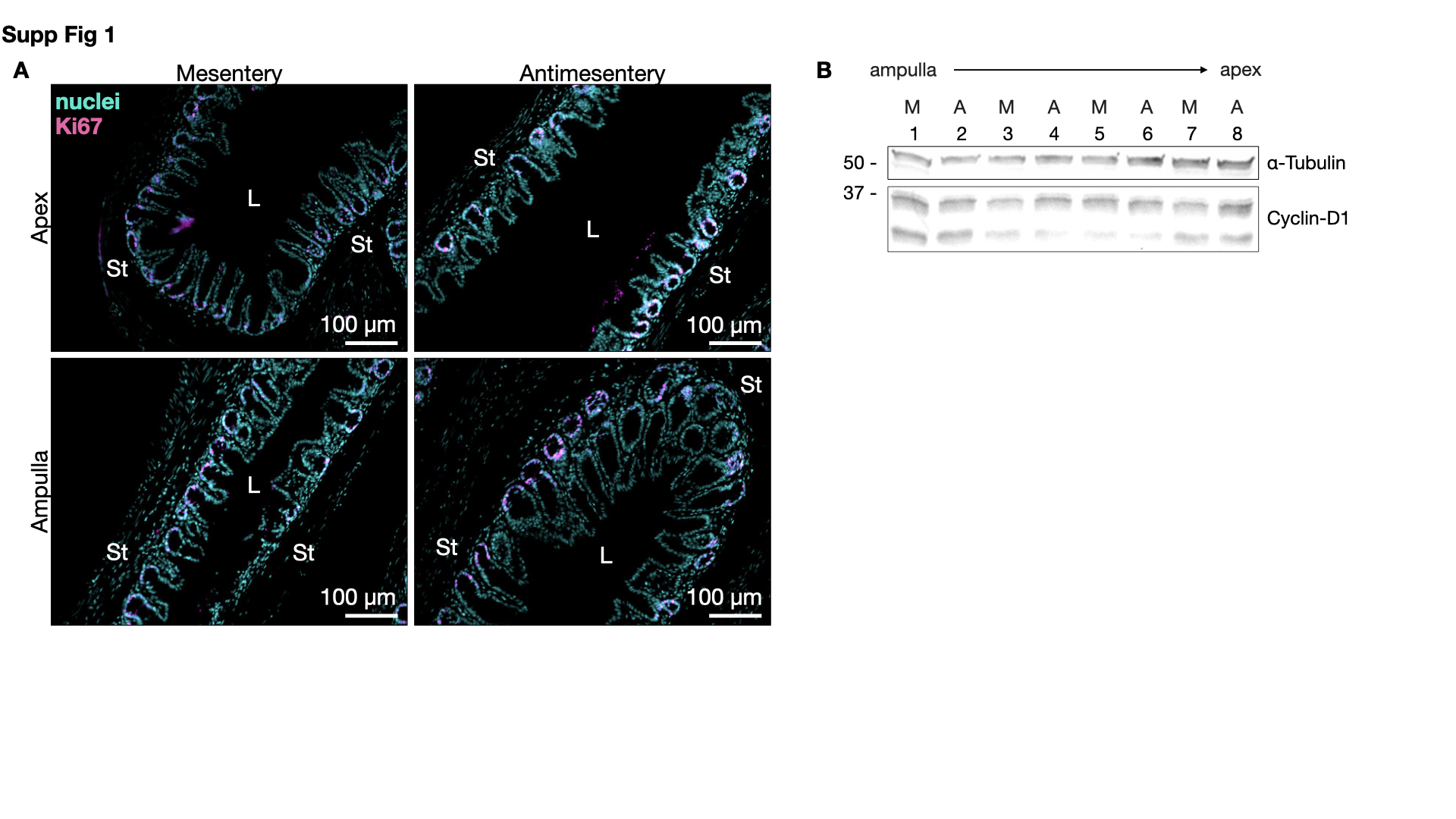

Supplement: Supplemental Material [file KGMI_A_2185029_SM6578.zip › CecAx ms Supp Fig1 021423.jpeg]

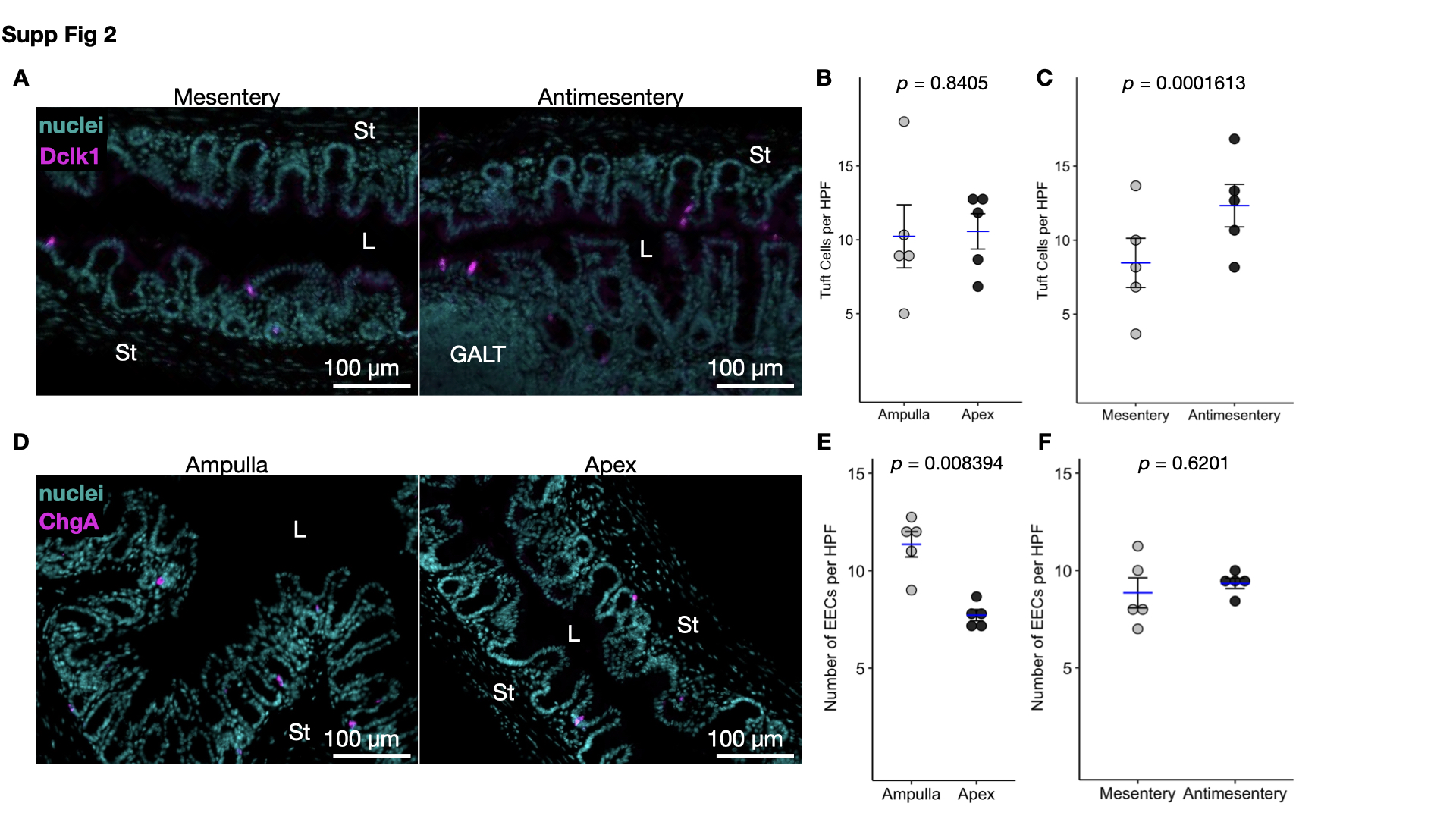

Supplement: Supplemental Material [file KGMI_A_2185029_SM6578.zip › CecAx ms Supp Fig2 021423.jpeg]
